# Combination of nutritional polyphenols supplementation with exercise training counteracts insulin resistance and improves endurance in high-fat diet-induced obese rats

**DOI:** 10.1038/s41598-018-21287-z

**Published:** 2018-02-13

**Authors:** Karen Lambert, Marie Hokayem, Claire Thomas, Odile Fabre, Cécile Cassan, Annick Bourret, Florence Bernex, Christine Feuillet-Coudray, Cécile Notarnicola, Jacques Mercier, Antoine Avignon, Catherine Bisbal

**Affiliations:** 1 PhyMedExp, University of Montpellier, INSERM U1046, CNRS UMR 9214. 34295, Montpellier, cedex 5 France; 2 Centre Hospitalier Régional Universitaire (CHRU) Montpellier, 34295 Montpellier, France; 3 UMR 866 (Dynamique Musculaire & Métabolisme) INRA, Place Viala, Montpellier, France; 4 University d’Evry Val d’Essonne, département STAPS. François Mitterrand Boulevard, 91025 Evry, France

## Abstract

Separately, polyphenols and exercise are known to prevent insulin resistance (IR) but their combined curative effects on established obesity and IR require further investigation. Therefore, we compared the metabolic effects of a combination of exercise and grape polyphenols supplementation in obese IR rats with high-fat diet (EXOPP) to the effect of high-fat diet alone (HF) or with a nutritional supplementation of grape polyphenols (PP) or with endurance exercise (EXO) during 8 wks. We observed an improvement of systemic and skeletal muscle insulin sensitivity in EXO and EXOPP rats. EXOPP rats compared to HF rats presented a lower insulinemia and HOMA-IR with higher liver and muscle glycogen contents. Interestingly, EXOPP rats had a 68% enhanced endurance capacity compared to EXO rats with also a higher activation of AMPK compared to sedentary and EXO rats with increased lipid oxidation. Together, our results suggest that grape polyphenols supplementation combined with exercise has a synergistic effect by increasing muscle lipid oxidation and sparing glycogen utilization which thus enhances endurance capacity. Our data highlight that in cases of established obesity and IR, the combination of nutritional grape polyphenols supplementation and exercise heighten and intensify their individual metabolic effects.

## Introduction

During the past decade, obesity has become an alarming worldwide epidemic mainly due to a preventable lifestyle of energy imbalance where the calories consumed outweigh energy expenditure. Physical inactivity and high-caloric diets trigger obesity and metabolic diseases associated with insulin resistance (IR) at the forefront of which lies type 2 diabetes (T2D)^1^. Thus, lifestyle interventions based on regular exercise and diet-induced weight loss programs have been found to reduce the risk of T2D. Interestingly, exercise alone or in combination with diet management is more effective in reducing T2D than metformin^2^, even after a 10-year follow-up^3^. Indeed, it is well-established that exercise training stimulates insulin sensitivity and alters glucose/lipid metabolism in skeletal muscle, liver and adipose tissue, favoring lipid oxidation^4,5^. Even though 50% of the beneficial effects of physical activity remain unexplained^6^, exercise is known to activate AMP-activated protein kinase (AMPK) pathway, leading to a rise in mitochondrial density and lipid oxidation^7^.

Interestingly, it has been observed that resveratrol, a dietary polyphenol mainly found in colored berries, is also able to activate the AMPK pathway, leading the scientific community to view it as an exercise-mimetic molecule^8^. However, resveratrol belongs to the polyphenols class of stilbenes, which is the less representative of total dietary polyphenols intake. There are more than 500 polyphenolic molecules found in edible plants (fruits/vegetables) in distinctive combinations of metabolites from different chemical classes^9^. The Mediterranean diet, naturally rich in polyphenols, has been associated with a reduction in IR, highlighting that the beneficial effects of polyphenols cannot be attributed to a single compound but rather to a synergistic molecular effect^10^. Indeed, grape polyphenols mixtures have been shown to reduce IR in rodents^11^, as well as in obese and T2D patients^12,13^. In addition, we previously demonstrated that 8-weeks of grape polyphenols supplementation in nutritional amounts was able to prevent fructose-induced IR in healthy volunteers with a high metabolic risk^14^. Although some studies have already investigated the beneficial effect of a combination of polyphenols (mostly from green tea) with exercise in healthy humans^15^ and rodents concomitantly subjected to a high-fat diet^16,17^, their results were contradictory. Indeed, some reported an improvement in oxidative metabolism or an increase in endurance capacity^17,18^ after polyphenols intake when other did not observe any notable effect^15,19,20^.

So, even if polyphenols and exercise have shown beneficial preventive effects on IR and obesity development, individually or in combination, data on their potential curative effect in combination are extremely scarce in established obesity. Skeletal muscle is an important metabolically active tissue that is pivotal to maintain whole-body homeostasis, due to its energy needs as well as its relatively large mass^1^. Skeletal muscle plays a central role in the beneficial effects of exercise and in glucose disposal^1^. Thus, the aim of our study was to investigate the potential curative effects on IR and skeletal muscle metabolism of a nutritional supplementation of grape polyphenols in combination with exercise compared to exercise and grape polyphenols individually, in high-fat diet obese and IR rats. Since it has been demonstrated that some polyphenols can mimic exercise, we also sought to investigate whether this combination could promote endurance capacity in established obese rats.

## Results

### Animal characteristics

**Figure 1:**
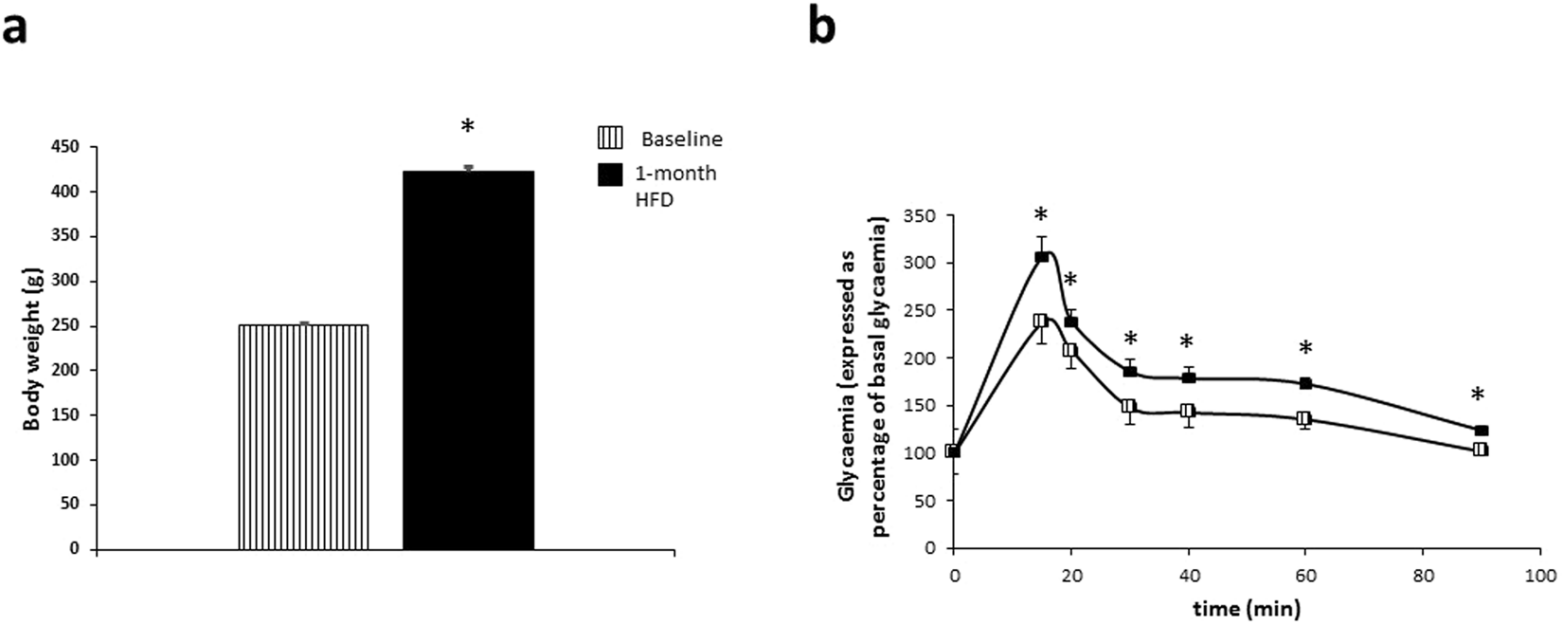
Animal characteristics after one month of high-fat diet. (**a**) Body weight and (**b**) glycaemia during glucose tolerance test expressed in percentage of basal glycaemia of 40 male Sprague-Dawley rats before (baseline, hatched) and after 4 weeks of an *ad libitum* high-fat diet (black). Data are expressed as mean ± SEM with n = 40 rats per group for (**a**) and n = 10 rats per group for (**b**). Significant difference: *p < 0.05 *vs* baseline.

After four weeks of high-fat diet (HF), rats presented an increase in both body weight (250.6 g ± 1.7 to 422.2 g ± 4.8, p < 0.001) and glycaemia during glucose tolerance test compared to baseline (Fig. 1a,b), as expected^21,22^.

**Figure 2:**
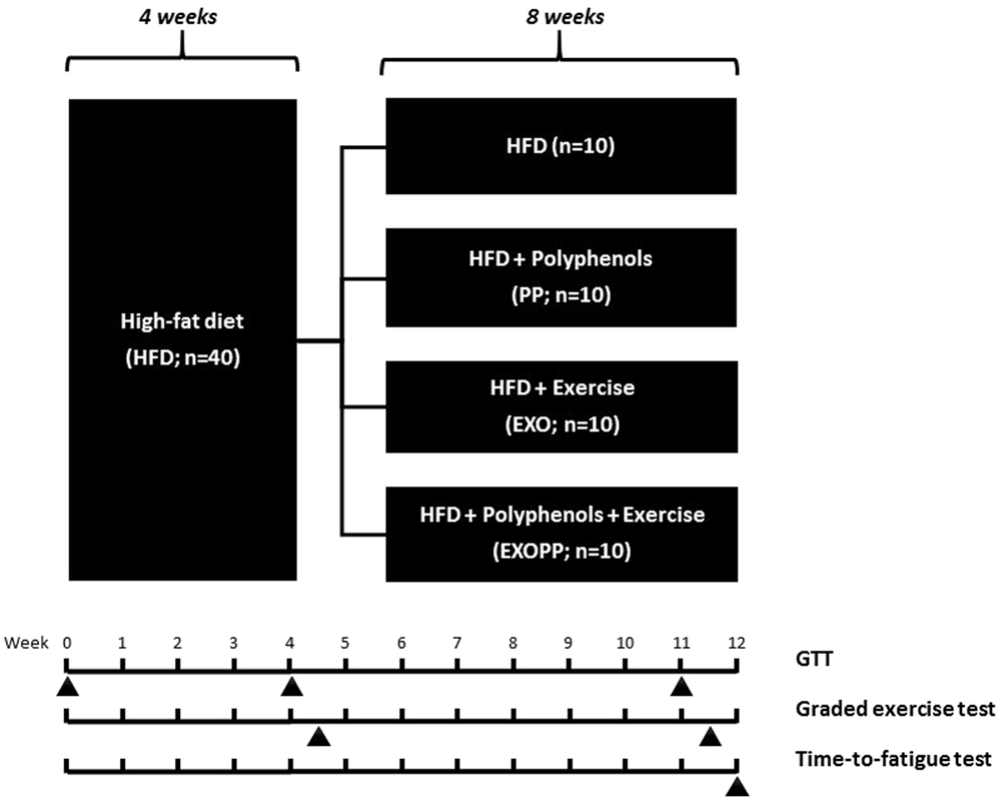
Experimental design of the study. 40 male Sprague-Dawley rats were fed a high-fat diet *ad libitum* for 4 weeks; then animals were divided into 4 groups (n = 10/group) for an additional 8-wk period with HFD alone (HF), HFD and supplementation with grape polyphenols extract at 50 mg/kg/d in 50 ml drinking water (PP), HFD and exercise 5 days/wk and 1 hr per day on a treadmill set to 32 m/min 10% slope (EXO), HFD and PP supplementation combined with exercise training (EXOPP). A glucose tolerance test was performed at arrival and after 4 and 11 wks. A graded exercise test was performed between the 4^th^ and the 5^th^ week and between the 11^th^ and the 12^th^ week. A time-to-fatigue test was performed at 12 weeks.

Then, during the eight following weeks, the animals were subjected to high-fat diet alone (HF) or HF with a supplementation of grape polyphenols extract at nutritional dose (PP) or to endurance exercise (EXO) or to the combination of exercise and polyphenols supplementation (EXOPP) (Fig. 2). Intake of the polyphenols mixture was 33.0 ± 2.1 ml/d for PP and 31.9 ± 1.7 ml/d for EXOPP groups compared to a water intake of 26.5 ± 1.3 ml/d for HF and EXO groups, indicating that the polyphenols mixture was well accepted by the animals.

### Combination of exercise and polyphenols supplementation improves anthropometric parameters

**Figure 3:**
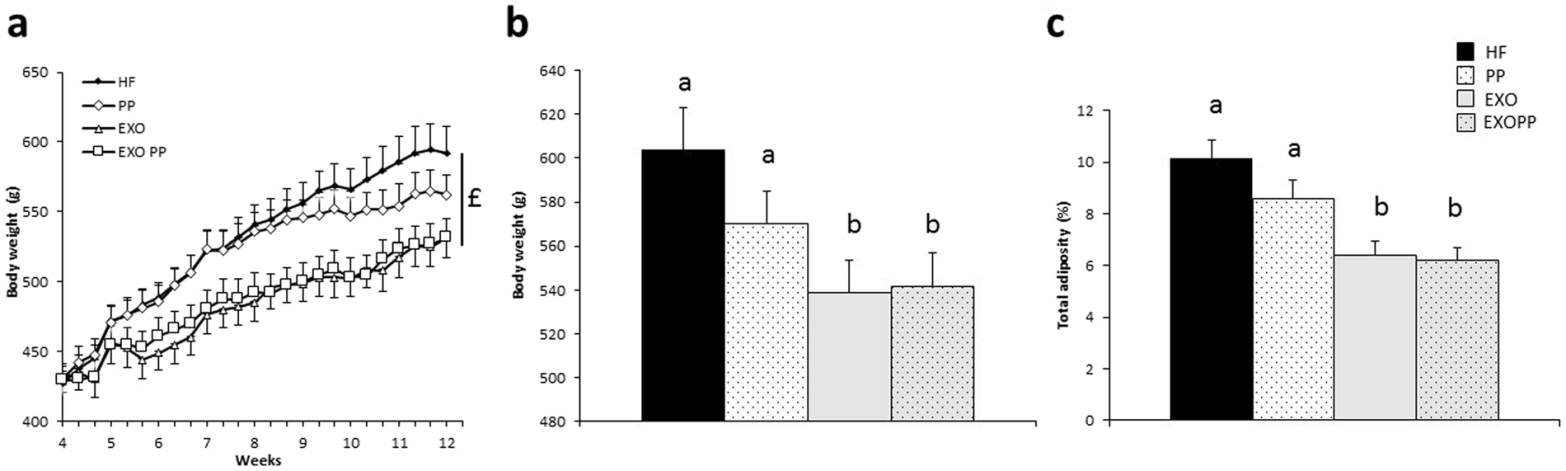
Anthropometric characteristics of rats. (**a**) Body weight gain from weeks 4 to 12. Body weight (**b**) and adiposity (**c**) after 12 weeks of intervention. Data are expressed as mean ± SEM with n = 8–10 rats per group. Bars not sharing a common letter are significantly different at P < 0.05. $ Training effect.

At the end of the intervention, weight gain (Fig. 3a,b, p < 0.01) and adiposity (Fig. 3c, p < 0.001) were lower in trained rats (EXO and EXOPP) compared to sedentary rats (HF and PP). EXOPP rats had lower weight gain and adiposity values but were not significantly different from EXO rats. Although they were sedentary, PP rats’ body weight was not significantly different from EXO or EXOPP rats’ body weight (Fig. 3a,b), whereas PP rats had greater adiposity than EXOPP rats (Fig. 3c). Trained rats had a higher muscle mass than sedentary rats (Table 1). EXOPP rats had the highest muscle mass determined by the *ratios* between extensor digitorum longus (EDL), soleus (SOL) and tibialis anterior muscles weight and total body weight (Table 1), although not significantly different from EXO rats (Table 1).

**Table 1:** Anthropometric parameters.

	HF	PP	EXO	EXOPP	Two-way ANOVA	Three-way ANOVA
Liver (g)	12.3 ± 0.5	12.3 ± 0.5	11.8 ± 0.6	11.6 ± 0.5	NS	NA
Liver/weight × 100	2.0 ± 0.1	2.2 ± 0.1	2.3 ± 0.1	2.1 ± 0.1	NS	NA
EDL/weight × 100	40.4 ± 2.6	50.4 ± 0.7*	50.0 ± 2.3*^$^	56.9 ± 1.7*^$^	E p < 0.001 S p < 0.001	E p < 0.05
SOL/weight × 100	42.2 ± 2.2	48.4 ± 2.4 *p = 0.073	50.7 ± 1.9*	52.1 ± 2.3*	E p = 0.011 S p = 0.098	S p= 0.002
Tibialis anterior/weight × 100	156.2 ± 5.7	166.9 ± 4.0	185.7 ± 4.5*	188.4 ± 4.6*^$^	E P < 0.001 S p = 0.186	

*p < 0.05 *vs* HF.

^$^p < 0.05 *vs* PP.

NS: Not significant.

NA: Not Applicable.

E: Exercise effect.

S: Polyphenols supplementation effect.

### Combination of exercise and polyphenols intake improves lipid parameters

Trained and polyphenols supplemented rats presented a lower leptin level compared to sedentary rats and rats without polyphenols (Table 2). EXOPP rats had the lowest leptin level, significantly different from PP (p < 0.05, d = 0.92) and HF (p < 0.01, d = 1.62) rats, but not from EXO rats (Table 2). Serum FFA concentration in EXOPP rats was lower compared to sedentary rats (HF and PP) only. Moreover, lipid profile was better in EXOPP rats, as indicated by the highest HDL levels (compared to HF rats: p < 0.01, d = 1.60) and lower atherogenic index (AI) than HF and EXO rats, suggesting that this latest effect was mainly due to polyphenols supplementation (Table 2, p < 0.05). Nonetheless, neither LDL/VLDL nor total cholesterol levels were affected in EXOPP rats, EXO and PP rats (Table 2).

**Table 2:** Plasma substrates and leptin levels determined in fasted condition.

	HF	PP	EXO	EXOPP	ANOVA
Leptin (ng/ml)	2.5 ± 0.4	1.6 ± 0.2*	1.4 ± 0.1*	1.2 ± 0.1*	Exercise and Supplementation p < 0.05
FFA (µM)	346.6 ± 77.1	218.9 ± 27.0	147.0 ± 31.6	174.7 ± 36.2	Exercise p < 0.05
HDL (mg/dl)	28.8 ± 1.8	31.1 ± 2.3	30.5 ± 2.3	36.7 ± 1.6*	Exercise p = 0.074 Supplementation p < 0.05
AI (cholesterol-HDL/HDL)	1.07 ± 0.2	0.74 ± 0.1	0.99 ± 0.1	0.74 ± 0.1	Supplementation p < 0.05
LDL/VLDL (mg/dl)	28.7 ± 2.7	24.5 ± 2.8	29.2 ± 3.0	26.1 ± 2.7	p> 0.05
Cholesterol (mg/dl)	62.8 ± 3.6	63.1 ± 3.3	57.7 ± 7.2	69.7 ± 4.3	p > 0.05
Insulin (µg/l)	1.6 ± 0.1	1.5 ± 0.1	1.4 ± 0.1	1.3 ± 0.1*^$^	Exercise p = 0.015 Supplementation p = 0.074
HOMA-IR	2.3 ± 0.1	2.2 ± 0.2	2.1 ± 0.2^$^	1.7 ± 0.1*^$^	Exercise p < 0.01

*p < 0.05 vs HF.

^$^p < 0.05 vs PP.

### Combination of exercise and polyphenols intake improves insulin sensitivity and glucose storage

**Figure 4:**
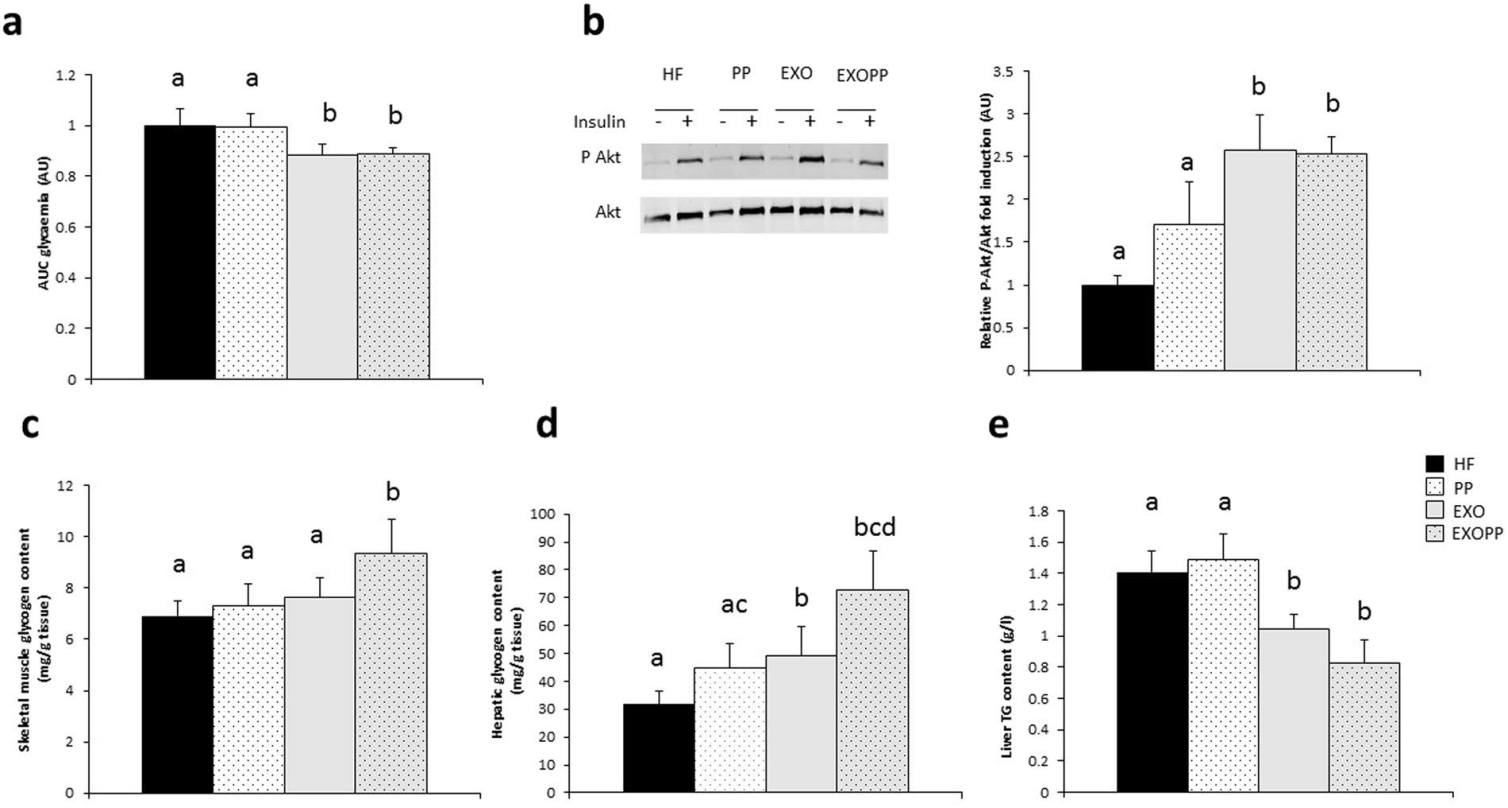
Measure of whole-body glucose homeostasis, skeletal muscle insulin sensitivity and glycogen content. (**a**) Area under the curve (AUC) for glycaemia. (**b**) P-Akt and Akt expression levels in soleus skeletal muscle, measured by immunoblotting after *ex vivo* insulin stimulation. A representative blot is shown. Quantification values are presented as P-Akt/Akt fold induction and are expressed relative to the HF value, which was set at 1. (**c**) and (**d**) Glycogen content in skeletal muscle (**c**) and liver (**d**). (**e**) Hepatic triglyceride content. Data are expressed as mean ± SEM with n = 8–10 rats per group. Bars not sharing a common letter are significantly different at  p < 0.05.

Whole-body glucose tolerance (Fig. 4a, p = 0.023) and muscle insulin sensitivity (Fig. 4b, p < 0.01) were improved with exercise, without effect of the supplementation. However, EXOPP rats had the lowest insulin level (compared to HF rats: p < 0.05, d = 1.27; compared to PP rats: p < 0.05, d = 1.99, exercise effect: p = 0.015) although not different from EXO rats’ insulin level (Table 2). Again, EXOPP rats had lower HOMA-IR index than HF (p < 0.05, d = 2.08), PP (p < 0.05, d = 1.53) and EXO (p = 0.057, d = 1.14), illustrating an improvement of insulin sensitivity. Given that the ability to store glucose in the form of glycogen is a hallmark of insulin sensitivity, we measured glycogen content in skeletal muscle and liver. When analyzed together, there is an increase in skeletal muscle and liver glycogen content due to exercise and in both tissues, glycogen content was higher in EXOPP group compared to HF group (Fig. 4c, p < 0.05, d = 0.83 and 4D, p < 0.05, d = 2.12). Hepatic glycogen content was also increase by polyphenols supplementation (Fig. 4d, p < 0.05). Moreover, hepatic triglyceride content was lower due to exercise and in EXOPP rats compared to HF rats (p = 0.01, d = 1.49) and PP (p < 0.01, d = 1.58) but not from EXO rats (Fig. 4e).

### Combination of exercise and polyphenols intake improves endurance capacity

**Figure 5:**
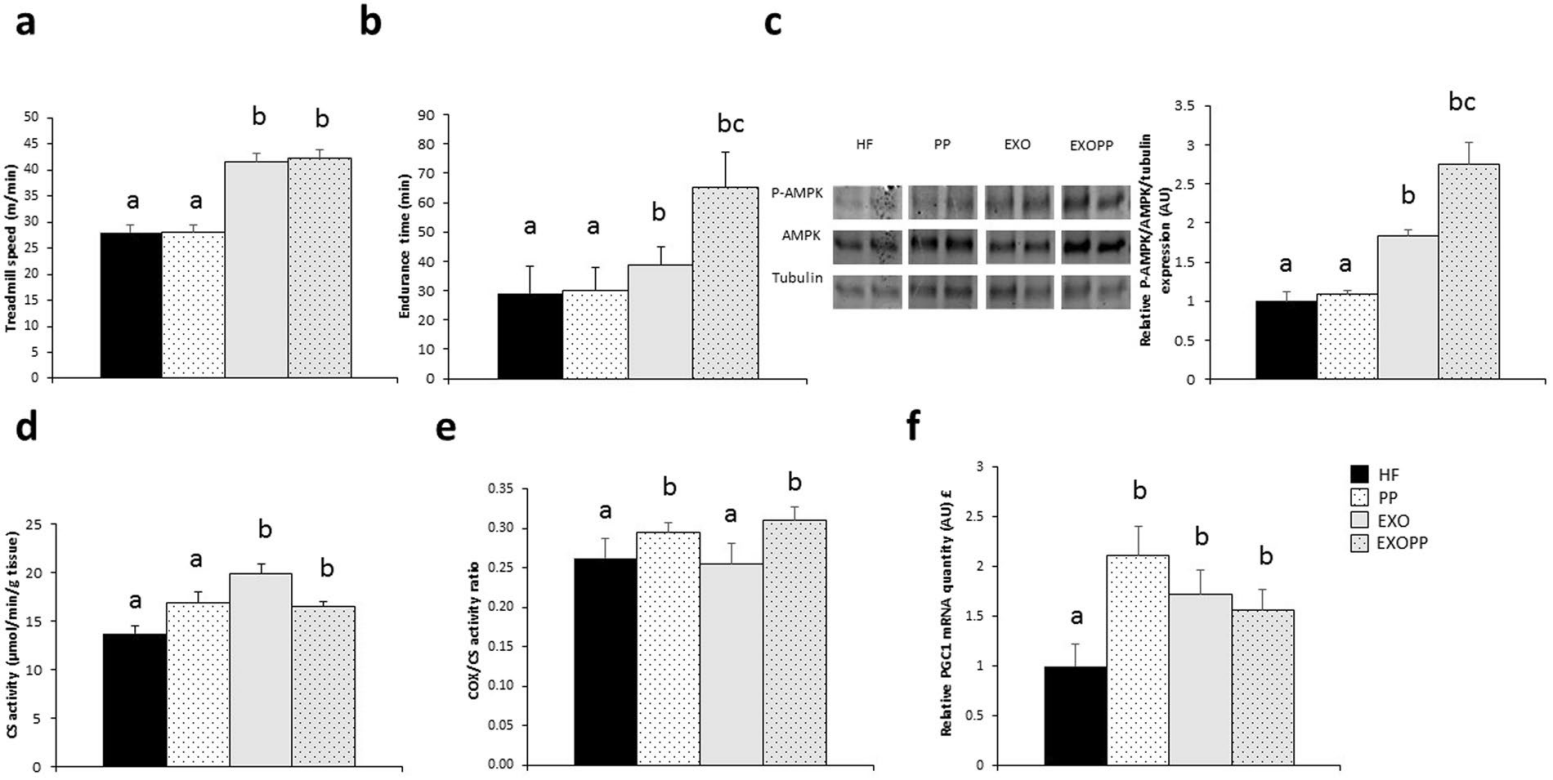
Measure of endurance and oxidative capacity. (**a**) Maximal speed and endurance time (**b**) on the treadmill. (**c**) P-AMPK, AMPK and tubulin expression levels in skeletal muscle, measured by immunoblotting. A representative blot of two rats by group is shown. Quantification values are presented as P-AMPK/AMPK *ratio* normalized to tubulin level, used as protein loading control and are expressed relative to the HF values, which was set at 1. (**d**) Mitochondrial CS and (**e**) COX/CS activities. Data are expressed as mean ± SEM with n = 8–10 rats per group. (**f**) PGC1α mRNA expression normalized by *RPL32* mRNA level and expressed relative to the HF value, which was set at 1. Data are expressed as mean ± SEM with n = 5–6 rats per group. Bars not sharing a common letter are significantly different at p < 0.05. £ interaction between exercise training and polyphenols supplementation.

After eight weeks of exercise, the increase of treadmill speed as endurance time were increase in trained rats compared to sedentary rats (p < 0.05). The increase of treadmill speed was dramatically greater in EXOPP rats compared to HF (p < 0.001, d = 4.67) and PP (p < 0.001, d = 4.25) rats (Fig. 5a). On the other hand, PP rats did not seem to benefit from the supplementation alone for endurance capacity. However, the noteworthy 68% increase in endurance time in EXOPP rats compared to EXO rats (Fig. 5b, p = 0.021, d = 1.03) illustrates a higher aerobic capacity when polyphenols were added to exercise training.

### Exercise and polyphenols supplementation increase oxidative capacity in skeletal muscle

**Figure 6:**
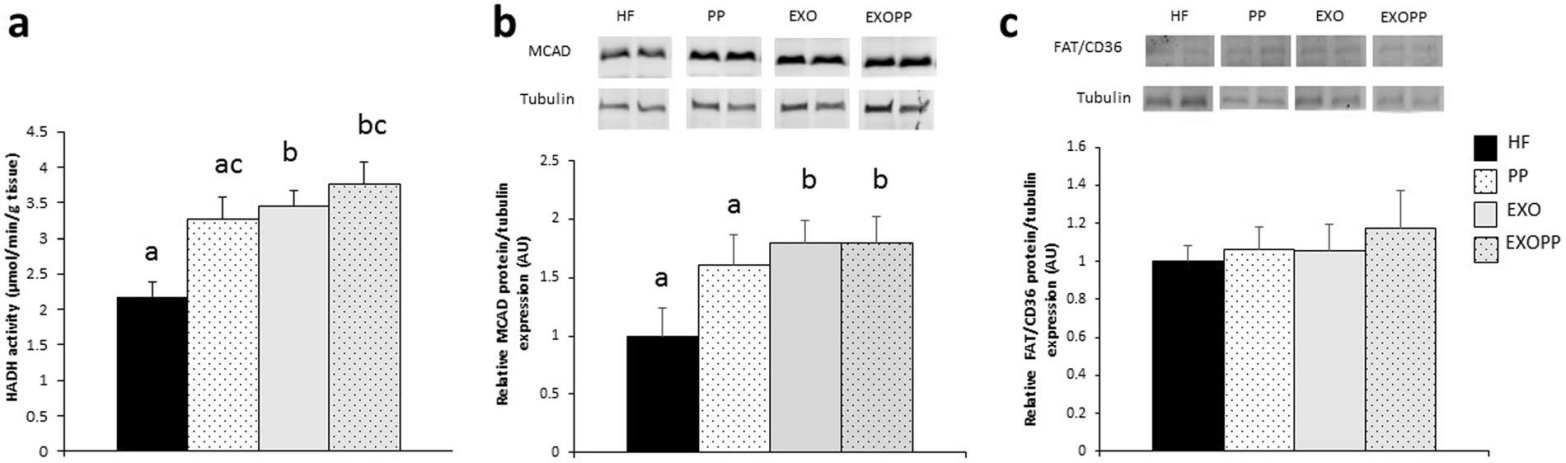
Measure of lipid oxidation. (**a**) Skeletal muscle HADH activity. MCAD **(b)** and (**c**) FAT/CD36 expression levels in skeletal muscle, measured by immunoblotting. A representative blot of two rats by group is shown. Quantification values are presented as MCAD (**b**) or FAT/CD36 (**c**) levels normalized to tubulin levels used as protein loading control and are expressed relative to the HF value, which was set at 1. Data are expressed as mean ± SEM with n = 8–10 rats per group. Bars not sharing a common letter are significantly different at p < 0.05.

Endurance capacity is related to oxidative pathway implying AMPK signaling and mitochondrial function^23^. We thus investigated AMPK activation by measuring its level of phosphorylation induced by eight weeks of exercise and/or polyphenols supplementation, as well as the activity of key mitochondrial enzymes such as citrate synthase (CS) and cytochrome oxidase (COX), and mRNA level of peroxisome proliferator-activated receptor gamma coactivator 1-alpha (PGC1α), a master gene in the regulation of mitochondrial biogenesis. P-AMPK/AMPK *ratio* was significantly higher in trained rats compared to sedentary rats and in EXOPP rats compared to HF (p < 0.05, d = 1.93), PP (p < 0.01, d = 0.31) and EXO (p = 0.054, d = 1.39) (Fig. 5c). Muscle oxidative capacity was also greater in EXOPP and EXO rats, as shown by the increase in CS activity (Fig. 5d, p = 0.048) in trained rats and in COX/CS enzyme activity *ratio* (Fig. 5e, p = 0.027) in polyphenol-supplemented rats only, compared to HF rats. PGC1α mRNA level was higher in all experimental groups compared to HF group (p < 0.05), suggesting an induction of mitochondrial biogenesis. Moreover, a statistical interaction between polyphenols supplementation and exercise (Fig. 5f, p < 0.013) would indicate that the effect of the supplementation is depending on the level of physical activity (sedentary *vs* trained rats). We also explored lipid β-oxidation with determination of hydroxyacylCoA deshydrogenase (HADH) activity, and protein level of medium-chain acyl-CoA dehydrogenase (MCAD). We also determined protein content of fatty acid translocase (FAT)/CD36, a main regulator of lipid transport. As an indicator of enhanced lipid oxidation, we observed higher HADH activity (Fig. 6a) in muscle from trained rats compared to sedentary rats but also higher HADH activity due to polyphenols supplementation. HADH activity was higher in muscle from EXOPP (p < 0.01, d = 2.42), EXO (p < 0.01, d = 2.23) and PP (p < 0.05, d = 1.50) rats compared to HF. Finally, we found an increase in MCAD protein content in EXOPP and EXO rats compared to sedentary rats (Fig. 6b, p < 0.05) while FAT/CD36 protein content was similar in all conditions (Fig. 6c).

## Discussion

Our study demonstrates that, in IR obese rats, combining exercise to a daily nutritional grape polyphenols mixture supplementation improves body composition, insulin sensitivity and endurance capacity.

While many studies reported the beneficial effect of exercise or polyphenols-enriched diets on metabolic parameters in obesity condition, only a few investigated the impact of the combination of these two different lifestyle interventions in rodents^16,17^. Importantly, most of these studies actually tested the ability of one or several different classes of polyphenols to prevent/attenuate development of obesity-induced metabolic alterations and not treat yet established obesity and metabolic alterations, since the supplementation started at the same time as the obesogenic high-fat diet. Here, polyphenols supplementation and exercise were imposed on rats after obesity and IR development to study their potential curative effect. The intake of the specific grape polyphenols mixture used in our study at the dose of 32–33 mg/kg/d is equivalent to nutritional amounts daily ingested by humans^9^. We show here that this nutritional polyphenols’ supplementation was sufficient to decrease rats’ body weight gain by 7%. In overweight humans at metabolic risk, a 5% decrease in body weight has been demonstrated to reduce the incidence of T2D by 58%^24^. Thus, a relatively modest weight lost is sufficient to provoke substantial metabolic improvements. Furthermore, polyphenols supplementation alone improved body composition since PP rats presented no significant difference in body weight compared to all others groups. Thus, it seems that in addition of a reduction of weight gain, PP rats also have the benefit of a part of the adaptations observed in EXO and EXOPP rats despite they were sedentary and not calorie-restricted. However, in human no data report such decrease in weight gain associated with polyphenols intake as previously found with exercise alone or combined with diet^25^. Interestingly, in polyphenols-supplemented rats (PP and EXOPP), we observed an increase in muscle mass, in accordance with our previous results in mice^26^ and others in menopausal women^27^. These data are in concordance with the exercise mimetic effect of polyphenols since they are able in resting condition to increase muscle mass as found with exercise^6^. Whatever the mechanism involved, muscle mass increase is a primary goal for clinical outcomes^28^ to fight against muscle atrophy^1^ and boost whole-body metabolism. In agreement with the literature^29,30^ our polyphenols supplementation was able to positively change body composition in obese rats. Importantly, the addition of an exercise program to our polyphenols supplementation in obese rats (*i.e*. EXOPP) exacerbated the reduction in body weight gain and adiposity. In normal rats, Dolinsky *et al*. found a significant reduction of body weight gain with exercise but not with resveratrol alone or in combination with exercise, despite a high dose of 146 mg resveratrol/kg/d^18^. Interestingly, in overweight males, only interval sprinting exercise (combined or not with green tea ingestion) induced a significant decrease in total body mass and increase in total lean mass compared to sedentary or green tea-consumer subjects^31^. The discrepancy between these results and our study could indicate that the beneficial effect of grape polyphenols supplementation on weight gain and muscle mass that we observed, could be related to the initial metabolic status of the population receiving polyphenols supplementation. Grape polyphenols supplementation would thus become beneficial only in challenged conditions like obesity.

Even though insulin sensitivity was better in exercise-trained rats, as assessed by the glucose tolerance test and muscle P-Akt/Akt *ratio*, our polyphenols mixture supplementation had no effect alone. However, the combination of polyphenols supplementation and exercise (*i.e*. EXOPP rats), resulted in lower insulinemia and HOMA-IR compared to HF rats, suggesting an improvement in insulin sensitivity. In overweight/obese women, exercise training at maximal fat oxidation associated with the consumption of five fruits and vegetables per day resulted in higher maximal lipid oxidation and insulin sensitivity^25^. These data corroborate our results using an animal model with nutritional intake of polyphenols. Another important mechanism participating in the improvement of insulin sensitivity is the increase of glycogen storage in insulin-sensitive tissues. It seems that our polyphenols supplementation alone enhanced liver ability to store glucose in the form of glycogen, as previously found with resveratrol^32^, leading to the improvement in insulin sensitivity, as previously demonstrated among rats consuming cinnamon polyphenols extract^33^. The combination of polyphenols and exercise (EXOPP) allowed an increase in liver and muscle glycogen content with a decrease in triglyceride hepatic content, which could favor insulin sensitivity.

Exercise and polyphenols intake have been shown to share the AMPK signaling pathway leading to the assumption that some polyphenols are “exercise-mimetics”^8,29,34^. Although exercise alone was able to activate both AMPK and mitochondrial enzymes, the combination of polyphenols and exercise (EXOPP) induced the highest AMPK activation suggesting a synergistic effect. The combination of polyphenols supplementation and exercise training also resulted in the highest endurance time, considerably increased in EXOPP compared to HF, PP and EXO rats. Thus, a synergistic effect was found for endurance time and AMPK activation when combining polyphenols supplementation with exercise training.

Interestingly, it is well known that aerobic capacity is inversely correlated to all-cause mortality^35^. Moreover, any increase in aerobic capacity is related to a decrease in all-cause and cardiovascular mortality^36^. Thus, the combination of polyphenols supplementation with exercise training could bring short-term benefits as improvement of insulin sensitivity and body composition but also potentially long-term benefits in terms of prevention of all-cause mortality. This greater aerobic capacity*, i.e*. endurance, in EXOPP rats due to a synergistic effect might be a consequence of better lipid oxidative capacity and mitochondrial function due to AMPK activation. Importantly, in human, lipid oxidation is associated with a metabolically healthy phenotype in overweight men^37^, improved whole-body lipid metabolism^38^ and insulin sensitivity^39^. In healthy human, the few studies that have investigated the combination of exercise and polyphenols supplementation^40–45^ frequently found an additive effect of moderate exercise and phytochemicals on lipid oxidation in resting condition, when only few studies reported deleterious effect on endurance^45^. In our study, CS and HADH activity and MCAD content were greater in muscle of trained animals. However, these parameters were not different between EXOPP and EXO rats, suggesting that the better endurance capacity of EXOPP group was not only related to lipid oxidation and mitochondrial function. Moreover, the discrepancy between PGC1α mRNA expression and mitochondrial function indicates a disconnection between mitochondrial function and biogenesis. In fact, the increase in lipid oxidation could be regulated in part, by carnitine palmitoyltransferase-1 activity, as previously found with proanthocyanidins supplementation^46^, through inhibition of acetyl-CoA carboxylase^47^.To enhance endurance capacity, it is important to increase fat oxidation while decreasing liver and muscle glycogen utilization. We hypothesized that the higher levels of hepatic and muscle glycogen content in EXOPP rats, concomitant with a lower hepatic TG content, could be due to an increase in fat oxidation as suggested by the increase in AMPK activation that we found, leading to spare glycogen storage^48^. Moreover, an increase in glycogen content has been previously found with other polyphenol^33^ and could be due to alteration in enzymes activities of glycogen synthesis pathway^32^ that could explain the increase in liver/muscle glycogen content we found in EXOPP group. As suggested by Fernandez *et al*. a large increase in hepatic glycogen content could be one determining factor for the increase of endurance capacity^49^ although others mechanisms could be involved^23^. Therefore, a reduction in glycogen utilization during exercise would enable to delay the onset of fatigue and thus increase endurance capacity^48,50^.

In conclusion, our study demonstrates that, in high-fat diet-induced obese rats, the combination of exercise and grape polyphenols supplementation counteracts anthropometric and metabolic impairments and increases endurance capacity, probably via enhancement in lipid oxidation and reduction in glycogen utilization.

Our data highlight that, when obesity and IR are already developed, the combination of nutritional grape polyphenols supplementation and exercise have synergistic metabolic effects, underlining the importance of both dietary and physical training recommendations in IR condition.

## Materials and Methods

### Ethical approval

All animal experimentation procedures were conformed to the Directive 2010/63/EU that was adopted on the 22^nd^ September 2010 for the protection of animals used for scientific purposes (agreement number: A34-172-38) and was approved by the Comité d’éthique en expérimentation animale Languedoc-Roussillon (CEEA-LR, C2EA-36) (protocol number: CEEA-LR-1062).

### Animals

Forty 6-wk old male Sprague-Dawley rats, obtained from Janvier Laboratories (St Berthevin, France) were placed, after acclimatization, in the facility, and fed *ad libitum* with a high-fat diet (HFD, D12330 Research Diets, containing 25.5% carbohydrate, 58% fat and 16.4% protein) for the 12 weeks duration of the experimental protocol. Rats were single-housed or double-housed in a temperature-controlled room and maintained with food and drink *ad libitum* in a 12:12 h light-dark cycle, lights on at 8:00 post-meridian. The training and *in vivo* tests were performed during rats’ dark cycle exposition. Body mass was monitored every two days throughout the experimental period.

### Experimental design

After 4 weeks of HFD, shown to induce skeletal muscle IR^21^, the animals were divided into 4 groups (n = 10/group) while simultaneously continuing the HFD for an additional 8-wk period (Fig. 2). Group specificities were the following: (i) HFD alone (HF), (ii) HFD and supplementation with grape polyphenols extract (PP) at 50 mg/kg/d per 50 ml of drinking water (PP), (iii) HFD and exercise training, 1 hr per day,/5 days/wk, consisting of running on a treadmill set at 32 m/min for a 10% slope (EXO), (iv) HFD and PP supplementation combined with exercise training (EXOPP). The extract of grape polyphenols used for supplementation (64.4% total polyphenols with 17.9% procyanidins, 4.3% anthocyanins and 533 ppm resveratrol) had been previously used^14,26^.

Glucose tolerance tests were performed on animals at the start of the protocol and then after 4 and 7 weeks of HFD. During the first 4 weeks, all animals were handled daily and familiarized 5 days a week/5–10 min/day with the treadmill set at 10 m/min at 10% slope. Graded exercise tests were performed after 4.5 weeks and at 11.5 weeks. The rats selected for the exercise groups (EXO and EXOPP) reached the required training intensity in 1 week. At 12 weeks, all animals were placed on the treadmill for a time-to-fatigue test (endurance test) (Fig. 2). Animals were euthanized 48 hrs after the last training session to avoid the acute effect of the last training session. After 12-hour fasting, animals were euthanized by exsanguination after isoflurane anesthesia. Immediately after death, a portion of liver, quadriceps and epididymal, perirenal, inguinal, and mesenteric adipose tissues were rapidly excised, weighed and directly frozen in liquid nitrogen. Adiposity (%) was calculated as the sum of the different adipose tissues (g) divided by rats’ total body weight and multiplied by 100. Soleus muscles were incubated in PBS with and without insulin 0.6 µM (Umuline RAPIDE, Lilly) at 30 °C for 15 min before snap-freezing in liquid nitrogen.

### Exercise tests

#### Graded exercise test (GET)

This test consisted in a progressive exercise test in which each rat initially ran at a speed of 10 m/min, up a 5% slope, for 3 min. Thereafter, the speed was increased by 3 m/min every 2 min until the rat was unable to keep pace with the treadmill belt despite encouragement to do so by application of manual solicitation at the hind legs.

#### Time-to-fatigue test (TtF)

This test consisted in a 3 min warm-up at 10 m/min, up at 15% slope, followed by an increase in speed to 25 m/min until exhaustion. A rat was considered as exhausted when it was no longer able to continue to run on the treadmill as judged by the rat spending >50% of the time or >30 consecutive seconds on the back of the treadmill despite encouragement.

### Blood analyses

#### Glucose tolerance test (GTT)

After a 12-hr fast, rats were injected intraperitoneally with a 30% glucose solution (2 g/kg body weight), as previously described^51^. Glucose level was determined at baseline and following glucose injection with a QuantiChrom^TM^ Glucose Assay Kit (BioAssay Systems) on blood collected from the tail, insulin level was determined at baseline using Ultrasensitive Rat Insulin ELISA (Mercodia). Homeostasis model assessment of IR (HOMA-IR) index was calculated using the formula: HOMA-IR = [fasting insulin (μIU/ml) × fasting glucose (mmol/ml)]/22.5.

#### Determination of free fatty acid (FFA), total cholesterol, low and very low-density lipoprotein (LDL/VLDL), high-density lipoprotein (HDL) and leptin plasma levels

Plasma from 12-hr-fasted rats were collected from the carotid artery of anesthetized animals and centrifuged for 10 min at 2,000 g. FFA, total cholesterol, LDL/VLDL, HDL levels were determined using respectively, EnzyChrom^TM^ Free Fatty Acid Assay Kit (EFFA, BioAssay System), EnzyChrom^TM^ LDL/VLDL Assay Kit and EnzyChrom^TM^ HDL Assay Kit (EHDL, BioAssay System). Atherogenic index (AI) was calculated as follow: Total cholesterol – HDL cholesterol/HDL cholesterol. Leptin concentration was determined by ELISA (BioVendor, C221111).

### Skeletal muscle and liver analyses

#### Skeletal muscle tissue homogenization and immunoblotting

Soleus samples were homogenized in an ice-cold lysis buffer containing 30 mM HEPES, 40 mM NaCl, 5 mM EDTA, 2 mM EGTA and 210 mM sucrose with phosphatase and protease inhibitors (Sigma-Aldrich), followed by a 10-min centrifugation at 10,000 g. Immunoblots were then performed as previously described^14^, using the following primary antibodies: phospho-Akt (P-Akt, Ser473), total Akt, phospho-AMPK (P-AMPK, Thr172), AMPK (all four from Cell Signaling Technology), FAT/CD36 and MCAD (both from Santa Cruz). Alpha-tubulin antibody (Sigma-Aldrich) was used for loading control.

#### Glycogen content

Portions of quadriceps and liver were used for glycogen content determination using the method described by Lo *et al*.^52^.

#### Citrate synthase (CS), cytochrome C oxidase (COX) and hydroxyacylCoA dehydrogenase (HADH) activity

Enzymatic activities were determined by spectrometry, as previously described on soleus homogenates^14^.

#### Triglyceride content in liver

Liver homogenates were mixed with Triton X-100 (0.1%), and TG were quantified spectrophotometrically by enzymatic colorimetric methods using commercially available kit: EnzyChrom™ Triglyceride Assay Kit (ETGA-200, BioAssay Systems).

### mRNA purification, reverse transcription (RT) and quantitative PCR (q-PCR)

Total RNA was isolated using TRIzol (Invitrogen, Thermo Fisher Scientific). To avoid genomic DNA contamination and amplification during PCR, RNA was treated with DNase-RNase free (Euromedex) before RT. RT was performed using the Verso cDNA kit (Thermo Scientific) in an incubation volume of 20 µl. Briefly, 5 µg of total RNA was denatured at 70 °C and then reverse-transcribed at 42 °C for 120 min following the manufacturer instructions. cDNA amplification was then performed by real-time q-PCR using the LightCycler® 480 SYBR Green I Master kit (Roche Diagnostics Limited) and specific primers for sequences of interest (*i.e. PGC1α*: forward CTACAGACACCGCACACATC, reverse CCTTTCAGACTCCCGCT; *RPL32*: forward CACCAGTCGGACCGATATGTGAAAA, reverse TGTTGTCGATGCCTCTGGGTTT) (Eurofins Scientific). q-PCR mix was composed as following: 2 µl of 1:10-diluted cDNA, 0.5 µl of each primer (0.5 µM final concentration), 5 µl 2× concentrated LightCycler® 480 SYBR Green I Master and 2 µl PCR Grade water for a final volume of 10 µl. Relative mRNA expression was quantified according to the comparative cycle threshold method, using Ct values in the formula 2^[Ct target gene – Ct reference gene]^ (2^ΔCt^) with *RPL32* as stable reference gene.

### Statistical analysis

Data are expressed as means ± standard error of the mean (SEM). Comparisons between treatment groups (effect of exercise (EXO + EXOPP) and effect of polyphenols supplementation (PP + EXOPP)) were performed using two-way analysis of variance (ANOVA) or a three-way analysis of variance (ANOVA) (effect of exercise, polyphenols supplementation and muscle type (EDL, SOL and tibialis anterior) after normality and equal variance have been tested. When the initial ANOVA has indicated significant differences between the tested groups, Bonferroni post-hoc tests of multiple comparisons versus high-fat group have been performed. Effect sizes were calculated using Cohen’s *d* with small, medium and large effects having the following values of 0.0 to 0.2, above 0.2 to 0.5 and above 0.5 respectively^31^. Statistical analysis was performed using SigmaStat3.5 software and the level of statistical significance was set at  p < 0.05. Bars not sharing a common letter are significantly different.

### Data availability

All data generated or analyzed during this study are included in this published article.
